# FOXC1 identifies basal-like breast cancer in a hereditary breast cancer cohort

**DOI:** 10.18632/oncotarget.12370

**Published:** 2016-09-30

**Authors:** Jeff Johnson, Michael Choi, Farnaz Dadmanesh, Bingchen Han, Ying Qu, Yi Yu-Rice, Xiao Zhang, Sanjay Bagaria, Clive Taylor, Armando E. Giuliano, Farin Amersi, Xiaojiang Cui

**Affiliations:** ^1^ Department of Surgery, Cedars-Sinai Medical Center, Los Angeles, CA 90048, USA; ^2^ Department of Pathology, Cedars-Sinai Medical Center, Los Angeles, CA 90048, USA; ^3^ Biostatistics and Bioinformatics Research Center, Cedars-Sinai Medical Center, Los Angeles, CA 90048, USA; ^4^ Samuel Oschin Comprehensive Cancer Institute, Cedars-Sinai Medical Center, Los Angeles, CA 90048, USA; ^5^ Department of Surgery, Mayo Clinic, Jacksonville, FL 32224, USA; ^6^ Department of Pathology and Laboratory Medicine, Keck School of Medicine, University of Southern California, Los Angeles, CA 90033, USA

**Keywords:** basal-like breast cancer, immunohistochemistry, BRCA, FOXC1, PARP inhibitor

## Abstract

Breast cancers arising in the setting of the hereditary breast cancer genes BRCA1 and BRCA2 are most commonly classified as basal-like breast cancer (BLBC) or luminal breast cancer, respectively. BLBC is an aggressive subtype of breast cancer associated with liver and lung metastases and poorer prognosis than other subtypes and for which chemotherapy is the only systemic therapy. Multiple immunohistochemical markers are used to identify the basal-like subtype, including the absence of estrogen receptor alpha, progesterone receptor, and human epidermal growth factor receptor 2. Forkhead box C1 (FOXC1) has been identified as a specific marker expressed in BLBC in general breast cancer cohorts. We examined an institutional cohort of breast cancer patients with germline BRCA1 (n=46) and BRCA2 (n=35) mutations and found that FOXC1 expression on immunohistochemical staining is associated with BRCA1 vs BRCA2 mutations [30/46 vs. 6/35]. In BRCA1 mutant tumors, FOXC1 was expressed in 28/31 BLBC tumors and 2/13 non-BLBC tumors, In BRCA2 mutant tumors, FOXC1 was expressed in 5/5 BLBC tumors and 1/30 non-BLBC tumors. In cell culture models of BRCA1-mutant breast cancer, FOXC1 is associated with increased proliferation and may serve as a marker for sensitivity to PARP-inhibitor therapy with olaparib.

## INTRODUCTION

Gene expression profiling with unsupervised clustering analysis has demonstrated distinct classes within the molecular heterogeneity of breast cancers. Subsequent studies have demonstrated that these molecular classes, which include luminal A, luminal B, Her2 expressing (HER2), and basal-like breast cancer (BLBC), have significant prognostic and predictive value [1–3]. Hereditary breast cancer arising in the setting of germline mutations in BRCA1 and BRCA2 is recognized to generally sort with the BLBC and luminal subtypes of breast cancer, respectively [3, 4]. While these molecular subtypes are defined by clustering analysis of gene expression profiles, in clinical practice these molecular subtypes are approximated by immunohistochemistry (IHC) and fluorescence in situ hybridization (FISH). In this classification system, tumors expressing estrogen receptor α (ERα) and/or progesterone receptor (PR) with low Ki-67 are categorized as luminal A; ER+ and/or PR+ with high Ki-67 or HER2+ are categorized as luminal B; ER-, PR-, and Her2+ by FISH are categorized as Her2+; and tumors lacking expression of these markers (ER-PR-HER2-) are “triple-negative” and categorized as BLBC [5]. This system has prognostic value and predicts response to specific endocrine or anti-HER2 therapy [6–10]. BLBC, which lacks ERα and HER2, has no recognized targeted therapy and has a relatively poor prognosis.

Additional IHC markers, such as epidermal growth factor receptor (EGFR) and basal cytokeratins, have been used to improve classification of BLBC, but these may not inform the molecular etiology of the disease and thereby may not serve as predictive markers for future therapy, and to some degree the inclusion of additional markers complicates the classification system and allows for discordant results [11–13]. The forkhead box transcription factor FOXC1 was identified in gene expression studies as a specific biomarker for BLBC. IHC expression of FOXC1 has been shown to be a specific marker for BLBC that has prognostic, even in cases of discrepancy between other IHC markers [12, 14]. Importantly, FOXC1 appears to play a functional role in BLBC, suggesting a potential role as a predictive marker for targeted therapies in development [14, 15].

Patients with germline mutations in BRCA1 have a significant risk of developing breast cancer by age 70, recently estimated at 69% (95% CI 56%-83%) [16]. Multiple studies have shown that 80-90%, of BRCA1 tumors are BLBC, as opposed to 10-15% of all tumors [2, 17, 18]. Conversely, approximately 20% of BLBC tumors show germline or somatic BRCA1 mutation [2]. This is in contrast to the second most common hereditary breast cancer, BRCA2-related breast cancer, which has a significantly different gene expression profile and is typically lower grade, is more differentiated, appears later in life, and belongs to the luminal/ER-positive subtype [4, 19]. Although both BRCA1 and BRCA2 have major functions tied to DNA repair through the homologous repair pathway, the specificity for BRCA1-related tumors to form BLBC suggests a role for BRCA1 in the regulation of genes related to that subtype. However, as patients with germline BRCA1 mutations represent a specific subclass of BLBC, it is yet to be established whether FOXC1 is also found within these tumors and whether there is a relationship between BRCA1/2 and FOXC1. In this study, we sought to demonstrate the clinicopathologic significance of FOXC1 expression in BRCA-associated breast cancer.

## RESULTS

### Clinicopathologic data and immunohistochemistry

**Figure 1:**
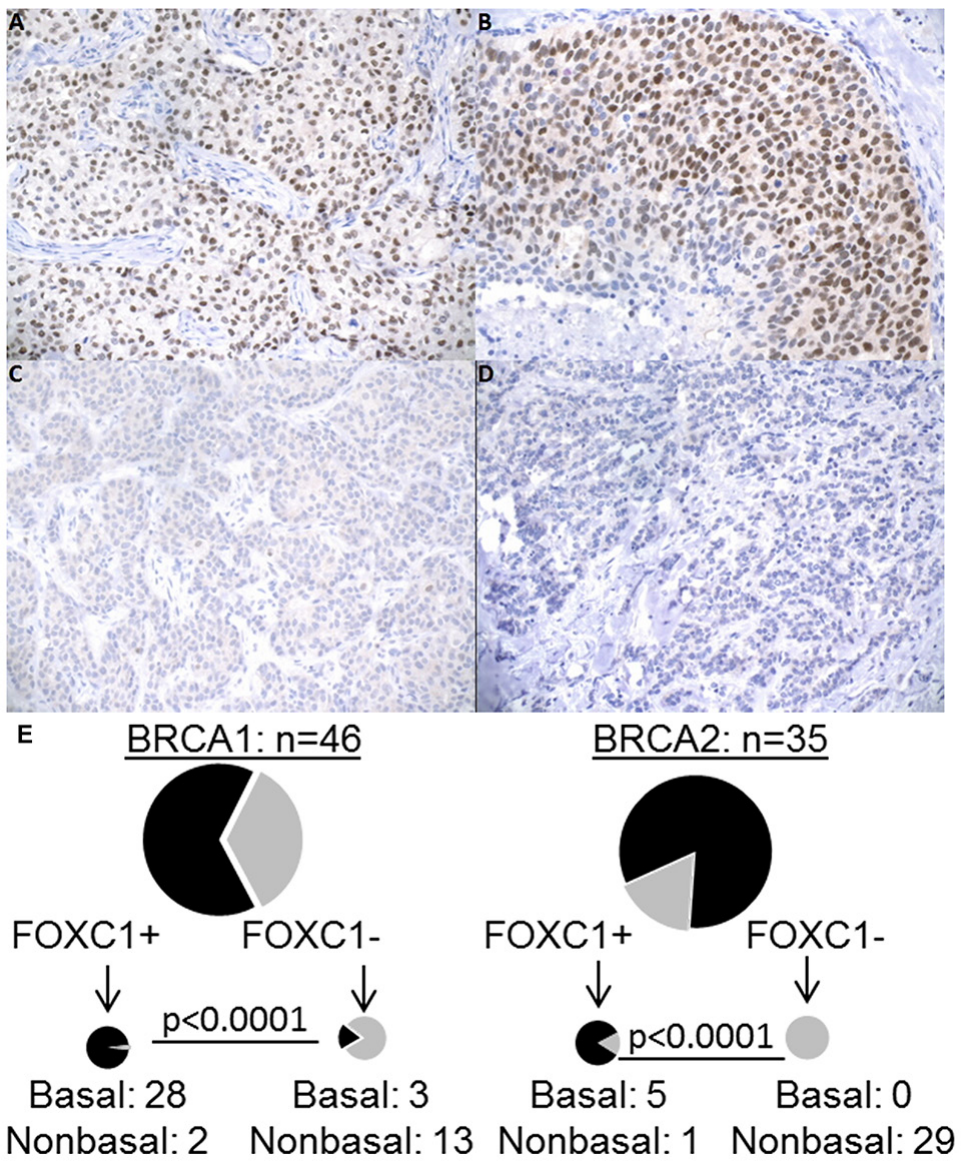
FOXC1 immunohistochemistry, BRCA1/2 mutation status, and molecular subtype **A.** BRCA1 mutant, basal molecular subtype with positive nuclear staining. **B.** BRCA wild-type, basal molecular subtype with strong nuclear positivity. **C.** BRCA2 mutant, luminal A molecular subtype with negative staining. **D.** BRCA wild-type, luminal A molecular subtype with negative staining. All images 200X. **E.** Distribution of BLBC/NonBLBC among FOXC1 IHC positive or negative cells for BRCA1 mutant and BRCA2 mutant tumors. BLCB: Basal-like breast cancer. NonBLBC: Non basal-like breast cancer, including Her2+ and Luminal A and Luminal B tumors.

Database review from two institutions identified 46 tumor samples from patients with germline BRCA1 mutations and 35 tumor samples from patients with germline BRCA2 mutations from 1995 to 2013 with available tissue for immunohistochemistry (IHC) staining. Paraffin-embedded tissue sections underwent IHC using a validated monoclonal FOXC1 antibody [14]. FOXC1 staining was considered positive if greater than 15% of cells demonstrated nuclear staining for FOXC1 (Figure 1). Available demographic and clinical information is noted in Table 1. FOXC1 association with the BLBC subtype was consistent with prior studies of BLBC demonstrating younger age of onset, higher tumor grade, and increased Ki67%. Also previously demonstrated, FOXC1-associated tumors had fewer lymph node metastases [20]. However, no significant differences were seen in the rates of distant metastases, distant recurrence, disease-free survival, or overall survival, although a trend was seen towards increased locoregional recurrence in FOXC1+ tumors (p=0.0512) (Supplementary Table S1). This may reflect relatively short follow-up times, a benefit of increased surveillance, early detection with lead-time bias, and/or more aggressive treatment among the BRCA-mutant population at our hospital.

**Table 1:** Clinicopathologic data for patients with BRCA mutation status and FOXC1 staining

Characteristic	FOXC1 + n (%)	FOXC1 − n (%)	P-value
**No. of patients**	36	45	
**Age at diagnosis (mean years ± SD)**	42.9 ± 13.3	50.7 ± 11.7	0.006*
**Tumor Size (mean mm ± SD)**	21 ± 9	20 ± 14	0.286**
**Tumor Grade**			<0.0001^†^
**1**	0 (0)	4 (100)	
**2**	3 (14)	18 (86)	
**3**	32 (58)	23 (42)	
**ER Positive**	4 (41)	41 (91)	<0.0001^‡^
**PR Positive**	3 (7)	38 (93)	<0.0001^‡^
**Ki67 % (mean ± SD)**	50.9 ± 21.9	19.9 ± 13.6	<0.0001**

* t-test.

** Mann-Whitney U test.

† Fisher's Exact test.

‡ Chi-Square test.

Figure 1 demonstrates the relationship between BRCA mutation status, FOXC1 staining, and molecular subtypes. FOXC1 staining was significantly associated with the basal-like phenotype defined by IHC markers (ER-PR-HER2-/EGFR+/CK5/6+): 92% (33/36) of FOXC1+ tumors were basal, and nearly all basal tumors (92%; 25/28), demonstrated FOXC1 staining (*p*<0.0001). Mutations in BRCA1 and BRCA2 were determined as part of patients' usual care with assays performed by Myriad Genetics (Salt Lake City, UT). Consistent with the associated subtypes, FOXC1 was significantly associated with the presence of BRCA1 germline mutation and absence of BRCA2 mutation (*p*<0.0007). Interestingly, 67% (31/46) of BRCA1 tumors were BLBC, a percentage lower than that reported in other studies [17]. Of the 46 BRCA1 tumors, 30 expressed FOXC1 and 28 of these were BLBC. Among the 35 BRCA2 tumors, six tumors expressed FOXC1 and five were BLBC. These findings demonstrate that FOXC1 is associated with BRCA1-mutant tumors and identifies the BLBC subtype in patients with germline BRCA1/2 mutations.

### Gene expression data

**Figure 2:**
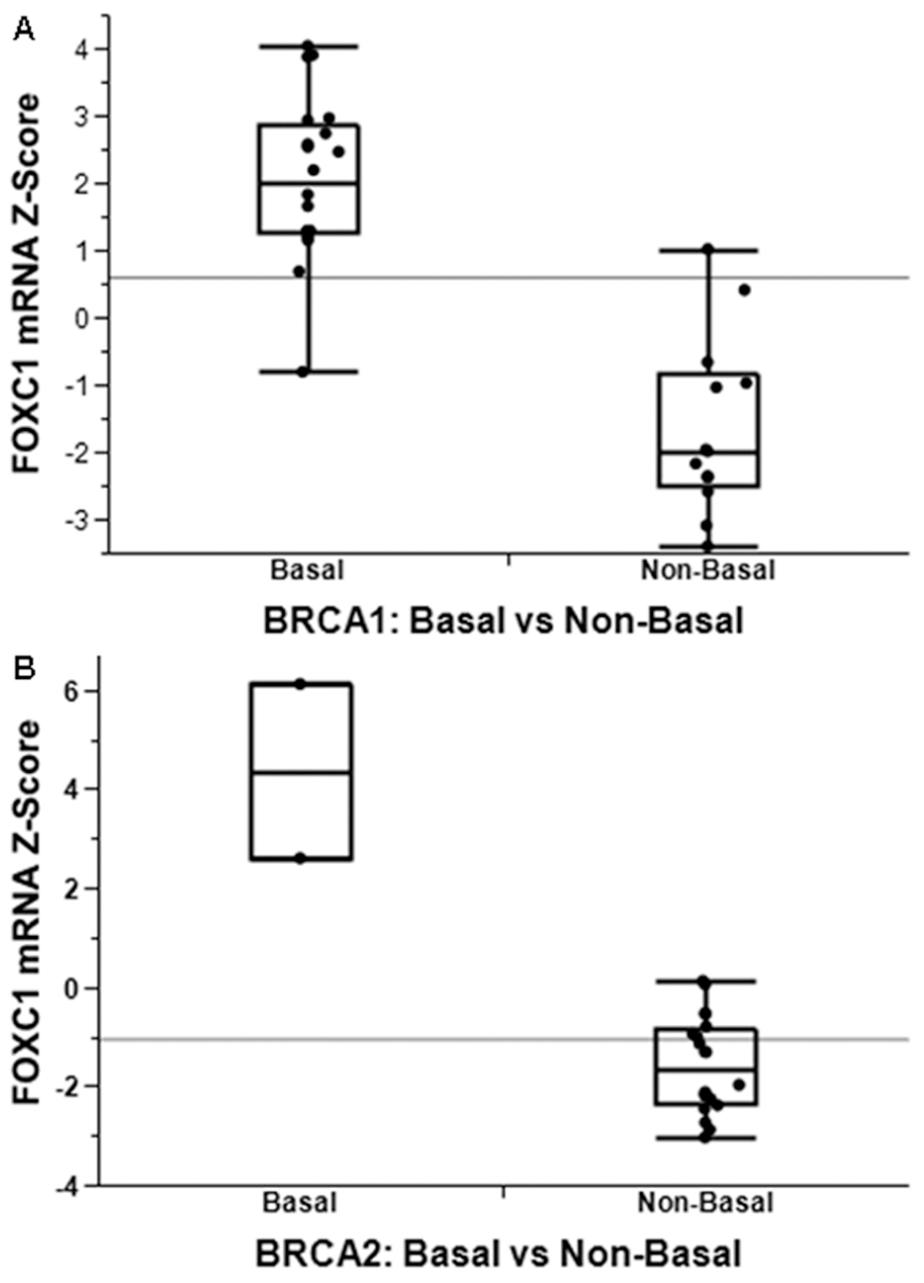
FOXC1 associated with BLBC with functional significance FOXC1 mRNA is significantly higher in basal-like breast cancer in both patients with both BRCA1 **A.** and BRCA2 **B.** mutations.

This association is also seen in mRNA expression data. Using the publicly available gene expression data from Larsen et al. (GSE40115), which includes gene expression profiling of 183 breast tumors, including 33 with BRCA1 germline mutation, 22 with BRCA2 germline mutation, and the remainder sporadic [21], we determined levels of FOXC1 between BLBC and non-BLBC for the BRCA1-mutant (Figure 2A) and BRCA2-mutant (Figure 2B) tumors. FOXC1 is markedly higher in BLBC compared to non-BLBC for both sets of tumors (p<0.0001).

### Cell proliferation and olaparib sensitivity

**Figure 3:**
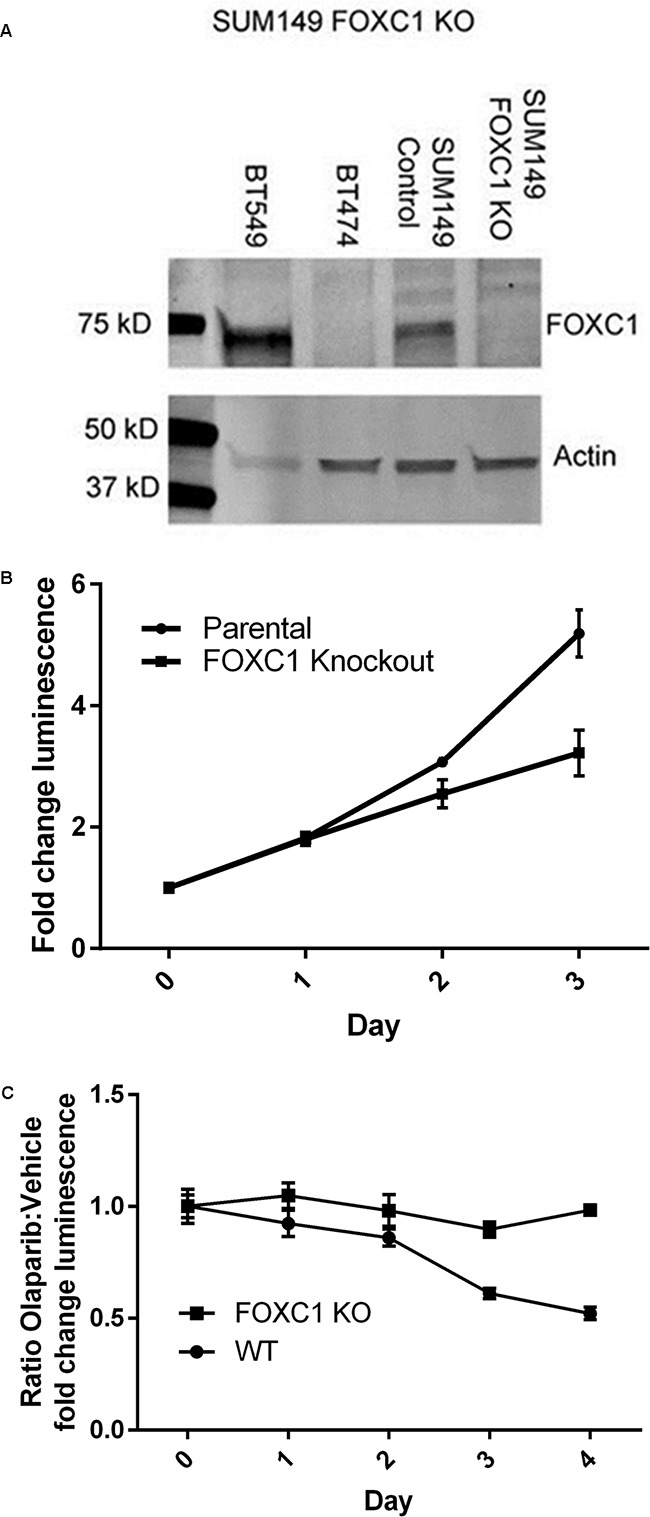
FOXC1 knockdown diminishes proliferation **A.** Western blot demonstrating CRISPR knockout of FOXC1 in SUM149. High-FOXC1 BLBC cell line BT549 and low-FOXC1 luminal cell line BT474 used as qualitative controls. **B.** FOXC1 knockdown results in diminished proliferation compared to FOXC1 wildtype in SUM149 cells. Mean ±SD for representative experiment with 6 technical replicates. Statistical significance for the difference in fold-change luminescence between parental and knockout cells, p<0.01 calculated by Mann-Whitney U test, reached on day 2 of growth. **C.** SUM149 FOXC1 wild-type cells are more sensitive to olaparib than FOXC1 knockout cells. Ratio of means ± combined SD for p<0.01 calculated by Mann-Whitney U test reached on day 3 of treatment.

**Figure 4:**
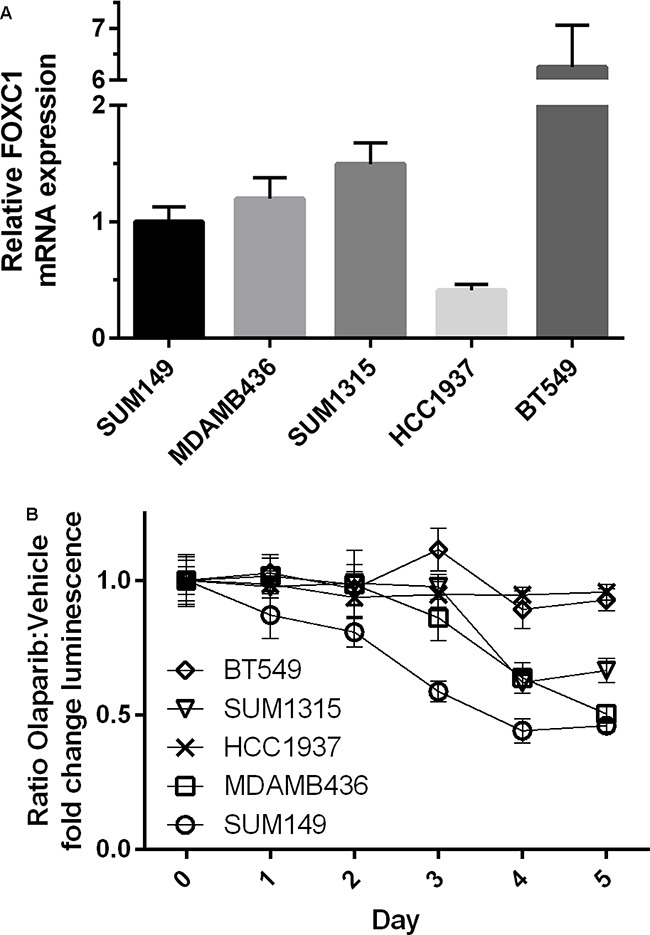
BRCA1-mutant cell line intrinsic FOXC1 level associated with PARPi sensitivity **A.** Relative FOXC1 mRNA expression for BRCA1-mutant cell lines and BRCA1 wild-type basal-like cell line BT549. Note Y-axis has been truncated for BT549 expression. BT549 FOXC1 expression significantly greater than all other cell lines (p<0.0001). HCC1937 FOXC1 expression significantly less than all other cell lines (p<0.01). **B.** Sensitivity of BRCA1 mutant cell lines and BT549 to olaparib. Ratio of means ± combined SD. p<0.01 calculated by Mann-Whitney U test reached on day 3 of treatment.

To determine whether FOXC1 has functional significance in BRCA1-mutant cells, we performed proliferation assays using control and CRISPR-mediated FOXC1-knockout (FOXC1-KO) SUM149 BRCA1-mutant BLBC cells (Figure 3A, Supplementary Figure S1), demonstrating decreased proliferation with FOXC1 knockout (Figure 3B). We also examined sensitivity of BRCA1-mutant cells to olaparib, a PARP inhibitor used in BRCA-mutant cancers to take advantage of synthetic lethality with the homologous repair defect. Control wild-type (WT) SUM149 cells were more sensitive to treatment with 10μM olaparib relative to DMSO vehicle than FOXC1-KO cells (Figure 3C). In examining the four available BRCA1-mutant BLBC cell lines and one BRCA1 wild-type cell line, we found that the BRCA1 mutant cell lines with relatively higher expression of FOXC1 demonstrated sensitivity to 10μM olaparib, whereas HCC1937, a BRCA1-mutant cell line with low FOXC1 expression, did not exhibit sensitivity (Figure 4B). BT549, a BLBC BRCA1 wild-type cell line with high FOXC1 levels (Figure 4A) also did not exhibit sensitivity to olaparib. Combined, these results suggest that FOXC1 is essential for BRCA1-mutant breast cancer cell growth and may predict sensitivity to treatment with olaparib in BRCA1-mutant cells, which warrants further validation using clinical samples.

## DISCUSSION

Prior work has demonstrated a number of genes specifically upregulated in BLBC, although the mechanism of the BLBC phenotype in BRCA1-mutant cancer is yet to be defined. We have demonstrated that FOXC1 can specifically identify BRCA1-mutant BLBC cancer. This and prior data suggests that the BLBC phenotype, and FOXC1 specifically can be regulated by BRCA1 [22]. In addition, the enrichment of FOXC1 and BLBC in BRCA1-mutant tumors may also be explained by development of cancer in FOXC1-expressing luminal progenitor cells, which are enriched in the setting of BRCA1 mutation [23, 24].

FOXC1 is known to play a critical role in embryonic ocular and brain development, with mutations or deletions resulting in glaucoma-related ocular dysgenesis and Dandy-Walker malformations [25]. Recently, FOXC1 has been demonstrated across a number of tissues to regulate the stem cell niche and stem cell activation/quiescence [26–29]. FOXC1 is also expressed in the developing mammary gland, but its exact function has not been described [23, 30]. FOXC1 is also being recognized in a wide range of cancers, including acute myeloid leukemia [31], hepatocellular carcinoma [32], renal cell carcinoma [33], gastric cancer [34], and non-small cell lung cancer [35].

In breast cancer, we have demonstrated that FOXC1 plays a role in proliferation in BRCA1-mutant BLBC cells, and prior studies have demonstrated a critical role for FOXC1 in the functional properties of BLBC, driving metastasis, epithelial-mesenchymal transition phenotype, and tumorigenesis [12, 36–39]. Our data shows that BRCA1-mutant SUM149 FOXC1 KO cells continue to proliferate, albeit at a reduced rate, suggesting that other FOXC1-independent pathways may be involved in the proliferation of these cells.

Further, FOXC1 is critical for EGFR-mediated cell proliferation and survival regulated via the ERK and PI3K/Akt pathways [40]. Downstream, FOXC1 have been shown to induce NF-κB signaling and induction of a SMO-independent Gli2 signaling pathway [15, 39]. Because of the critical role of FOXC1 in BLBC cell function, along with an opposing expression pattern between BRCA1- and BRCA2-mutant breast cancers, it is speculated that BRCA1, not BRCA2, mutation-elicited signaling might synergize with FOXC1 action in mammary tumorigenesis. One possible implication of this finding is heterogeneity in the phenotype, and possibly the cells of origin, of germline BRCA-mutation associated tumors. The mammary epithelium is sorted into subgroups representing different stages in the differentiation of tissue from mammary stem cells to one of the mature downstream pathways, basal myoepithelial cells or mature luminal cells [41]. Multiple studies suggest that BRCA1 is essential for the differentiation and maturation of luminal cells, and that BRCA1 mutation may lead to an arrest in differentiation and increase in the number of stem and/or progenitor cells [24, 41, 42]. This is significant as the different molecular subtypes have been correlated with different cells of origin along, with BLBC associated with luminal progenitor cells and luminal cancers associated with mature luminal epithelial cells [24, 43]. The presence of FOXC1, which was shown to be induced in luminal progenitor cells, suggests that germline BRCA mutations may cooperate with FOXC1 to induce tumorigenesis in the BLBC subgroup of BRCA-mutant breast cancer.^21^ The heterogeneity demonstrated especially in BRCA1 associated tumors may affect therapeutic options and requires further investigation.

This study also demonstrates that expression of FOXC1 in BRCA1 mutant cell lines correlates with sensitivity to olaparib. Whether this is due to rates of proliferation or another mechanism is yet to be explored, but this, and the specificity of FOXC1 in BRCA1-mutant tumors, suggests a possible role for FOXC1 as a marker for targeted therapy. Certainly the BRCA1-FOXC1 axis deserves further attention as a mechanism for the etiology of BLBC, and ultimately may prove FOXC1 as a marker for targeted therapies against BRCA1 and/or BLBC.

## MATERIALS AND METHODS

### Immunohistochemistry

Data on molecular subtype as well as ERα, PR, Her2, and Ki-67 was collected from pathology reports from surgically resected tumor specimens. IHC of ERα, PR, Her2, Ki-67, EGFR, and CK5/6 was performed as part of routine surgical pathology of tumor specimens. IHC staining and interpretation was performed consistent with guidelines published by the American Society of Clinical Oncology/College of American Pathologists [44]. Tissue sections demonstrating any intensity of ER or PR staining (low, moderate, high) in ≥1% of cells were reported as “positive.” Ki-67 is reported as a percentage of cells demonstrating nuclear staining, and tumors with ≥20% of cells staining positive are interpreted as “high.”[45] Tumors expressing estrogen receptor α (ERα) and/or progesterone receptor (PR) with low Ki-67 were categorized as luminal A; ERα + and/or PR+ with high Ki-67 or HER2+ were categorized as luminal B; ERα -, PR-, and Her2+ by FISH were categorized as Her2+; and tumors lacking expression of ERα, PR, and HER2 with expression of EGFR and CK5/6 were categorized as BLBC [5, 13, 46]. FOXC1 status was determined by staining of archived formalin-fixed paraffin-embedded breast cancer tissue from surgical specimens using a validated monoclonal FOXC1 antibody (Onconostic Technologies, Inc., Champaign, IL).^12^ Two independent pathologists blinded to BRCA status assessed slides for nuclear staining of FOXC1 and determined the percentage of positive cells. Sections demonstrating positive nuclear staining in ≥15% of cells were deemed positive for FOXC1 staining.

### Gene expression arrays

Data from Larsen et al. (GSE40115) was obtained from the Gene Expression Omnibus [21]. Z-scores for gene expression levels were compared with the Mann-Whitney U test.

### Proliferation assays

Cells were seeded at 1 × 10^3^ cells/wellin 96 well plates. SUM149 cells were cultured in Ham's F12 nutrient mixture, 5% fetal bovine serum, and 1% penicillin/streptomycin, supplemented 0.01 mg/ml insulin and 500 ng/ml hydrocortisone [47]. Luminescence, based on quantity of ATP present, was used a readout for measuring number of viable cells and was determined every 24 hours using CellTiter-Glo Assay (Promega) according to manufacturer protocols. Daily luminescence values were normalized to Day 0 and compared using Mann-Whitney U test. A single experiment included six technical replicates and all experiments were repeated three times. Data shown are mean±SD from a representative experiment.

### Drug sensitivity

Cells were seeded at 1 × 10^3^ cells/well (BT549), 2 × 10^3^ cells/well (SUM1315, HCC1937), 3 × 10^3^ cells/well (SUM149), or 4 × 10^3^ cell/well (MDA-MB-436) in 96 well plates. Cells were cultured according to previously published protocol [47]. Cells were treated 24 hours after plating with 1μM olaparib in DMSO or equivalent volume of DMSO. Relative cell number was assessed with CellTiter-Glo Assay (Promega) every 24 hours. Daily luminescence values were normalized to Day 0 and are expressed as the ratio of mean values for luminescence for olaparib treated/DMSO treated cells for each cell line on a given treatment day ±combined SD. A single experiment included six technical replicates and all experiments were repeated three times. Data are from a representative experiment. Daily luminescence values were compared using Mann-Whitney U test.

### FOXC1 knockout cells

The knockout of FOXC1 in SUM149 cells was performed according to the protocol published previously [48]. Briefly, the single guide RNA (sgRNA) for knocking down FOXC1 was designed by an online tool (http://crispr.mit.edu/). The sequence of the sgRNA is 5'-GGGTGCGAGTACACGCTCAT-3'. The sgRNA and its complementary strand were synthesized (ThermoFisher), annealed, and then subcloned into LentiCRISPRv2 vector (Addgene). The cells were infected with LentiCRISPRv2-FOXC1 and then selected with puromycin for 48 hours. The knockout efficiency was evaluated by western blotting.

### qRT-PCR

Total RNA was extracted and reverse transcription performed using the RNeasy Mini Kit (Qiagen) the QuantiTect Reverse Transcription Kit (Qiagen) according to manufacturer's instructinos. Quantitative PCR was done using an iCycler iQ Real-Time Thermocycler (Bio-Rad Laboratories) [15]. GAPDH was used as an internal control. FOXC1 primers used were: 5'-catccgccacaacctctcgct-3' (forward) and 5'-gtgcagcctgtccttctcctcc-3' (reverse). Fold change was calculated with delta delta Ct value. Bar graphs represent mean±SEM of three independent experiments with three technical replicates. Pairwise comparisons analyzed with Tukey's HSD.

### Statistics

Data were analyzed as described using GraphPad Prism version 6 for Windows (GraphPad Software, La Jolla, CA USA) or JMP version 12 for Windows (SAS Institute, Inc., Cary, NC USA). For all statistics, p<0.05 was used for statistical significance. For clinicopathologic variables, normally distributed variables were compared with 2-tailed t-test and non-normally distributed variables with the Mann-Whitney U test. Fisher's Exact test was used for mean comparisons between >2 groups. Chi-Square test was used to compare proportions.

## SUPPLEMENTARY FIGURE AND TABLE


