# Supplementary material for: FOXC1 identifies basal-like breast cancer in a hereditary breast cancer cohort
Source: Oncotarget. 2016 Sep 30;7(46):75729–38. doi: 10.18632/oncotarget.12370 (PMC5342773; doi:10.18632/oncotarget.12370)
Supplement: Supplementary file 1 [file oncotarget-07-75729-s001.pdf]

FOXC1 identifies basal-like breast cancer in a hereditary breast cancer cohort

SUPPLEMENTARY FIGURE AND TABLE

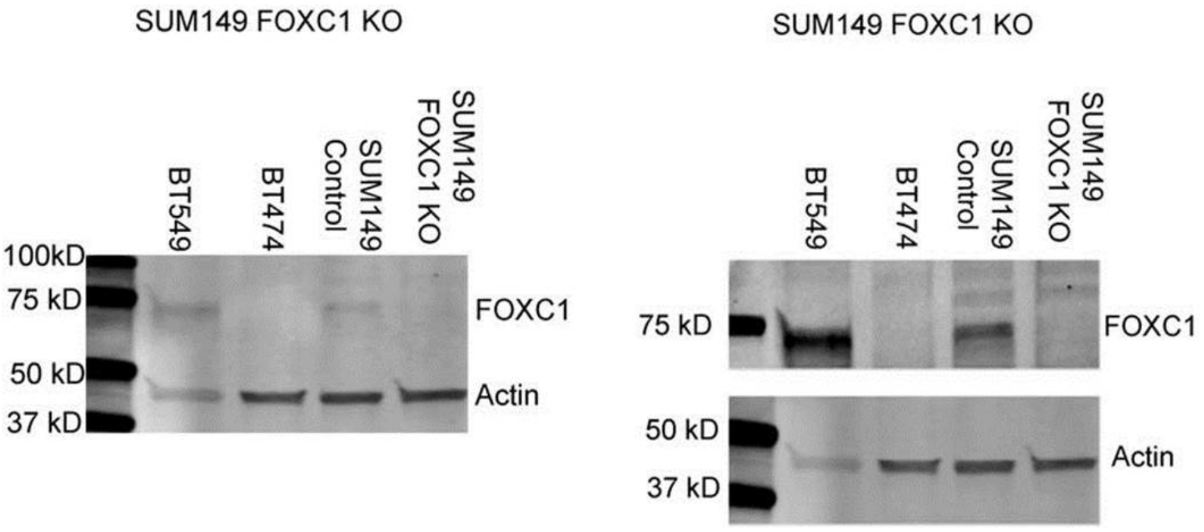

Supplementary Figure S1: Western blot demonstrating CRISPR knockout of FOXC1 in SUM149. High-FOXC1 BLBC cell line BT549 and low-FOXC1 luminal cell line BT474 used as qualitative controls. Left panel demonstrates uncut membrane. Due to relatively weak signal of FOXC1 relative to actin, the right panel was generated with increased exposure and vertical expansion to clearly delineate the FOXC1 band.

Supplementary Table S1: Available clinical data for patients with BRCA mutation status and FOXC1 staining, n=72

	FOXC1 + n =26	FOXC1 − N=45	P-value
**# of positive LN (mean ± SD)**	0.1 ± 0.5	2.6 ± 5.6	0.009**
**LN Positive**	9 (30)	21 (70)	0.081‡
**Stage**			0.6903†
1	12 (39)	19 (61)	
2	12 (44)	15 (56)	
3	2 (25)	6 (75)	
**Distant Metastasis**	0 (0)	1 (100)	1†
**Locoregional Recurrence**			0.0512
No	24 (35)	45 (65)	
Yes	3 (100)	0 (0)	
**Distant Recurrence**			0.644
No	26 (39)	41 (61)	
Yes	1 (20)	4 (80)	
**Disease-Free Survival (mean days ± SD)**	2397 ± 2226	2017 ± 1437	0.948
**Overall Survival (mean days ± SD)**	3049 ± 2830	2330 ± 1741	0.958

LN: Lymph nodes. *t-test. ** Wilcoxon-Mann-Whitney test. †Fisher's Exact test. ‡Chi-Square test.
